# Nucleolin Alterations and ROS Production Associate With Sensitivity of AML Cells to Venetoclax

**DOI:** 10.1096/fj.202504934RR

**Published:** 2026-09-26

**Authors:** Kateřina Wolfová, Petra Otevřelová, Barbora Brodská

**Affiliations:** ^1^ Department of Proteomics Institute of Hematology and Blood Transfusion Prague Czech Republic

**Keywords:** acute myeloid leukemia, Bcl‐2, Decitabine, Doxorubicin, nucleolin, ROS, Venetoclax

## Abstract

Acute myeloid leukemia (AML) is a heterogeneous disease with large spectrum of specific mutations and gene aberrations. Recently, the Bcl‐2 inhibitor Venetoclax, in combination with hypomethylating agents (HMAs), was approved for older (> 65 years) AML patients, as well as for those unfit for intensive induction chemotherapy. In addition to Bcl‐2 inhibition, Venetoclax also induces generation of reactive oxygen species (ROS). We demonstrated that distinct fraction exhibiting specific features arises during 24 h of sample exposure to Venetoclax. This fraction displays characteristic preapoptotic markers as mitochondria depolarization and partial Annexin V surface positivity. Moreover, monitoring of ROS showed negative correlation between signals detected using H_2_DCFDA and CellROX probes pointing to dynamic ROS changes induced by Venetoclax. The addition of HMA (Decitabine) had almost no effect on cell viability or ROS production but caused proliferation arrest in sensitive cells. In our panel of AML cell lines and primary AML samples we have found a correlation between ROS production, markers of apoptosis, and attenuation of Bcl‐2 activity after Venetoclax treatment. Level of Mcl‐1, another antiapoptotic protein from the Bcl‐2 family, was reduced in sensitive cells, but increased in the resistant samples in response to Venetoclax. Moreover, the nucleolar protein nucleolin (NCL), which is frequently overexpressed in AML cells, was significantly deregulated in Venetoclax‐treated cells. In particular, both NCL protein level and specific phosphorylation decreased in fractions sensitive to Venetoclax. Our findings suggest that Venetoclax targets distinct cell subpopulation, and that ability of a cell to follow increased ROS drives its response to Venetoclax.

## Graphical abstract

Venetoclax‐induced Bcl‐2 inhibition corresponds with Mcl‐1 attenuation and decrease of Nucleolin (NCL) level and activity in sensitive cells. Simultaneously, distinct cell subpopulations with opposite mitochondrial (CellROX label, magenta) and cellular (H_2_DCFDA stain, green) ROS occur, and the H_2_DCFDA‐positive population shows markers of early apoptosis together with low NCL. Conversely, Venetoclax‐resistant cells upregulate the Mcl‐1 level and NCL phosphorylation, while levels of phospho‐Bcl‐2, NCL, and ROS content remain unchanged.

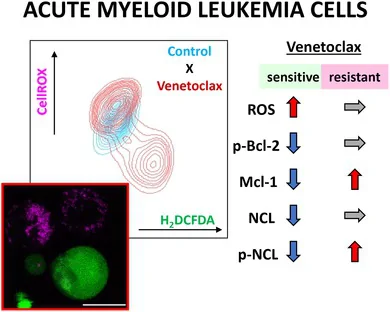

## 1 Introduction

Acute myeloid leukemia (AML) is an oncohematologic disorder, which is very heterogeneous in its characteristics and manifestations. Despite therapeutic advances, AML remains challenging to treat and generally has a poor prognosis, especially in patients unfit for intensive chemotherapy (IC) [1]. IC is based on the 3 + 7 (Cytarabine + Anthracycline) induction regimen and subsequent consolidation followed by hematopoietic stem cell transplantation [2]. Venetoclax (ABT‐199) can be added to the drugs used in IC [3, 4]. Venetoclax belongs to BH3‐mimetics and it specifically targets Bcl‐2 protein [5]. It has excellent efficacy even as monotherapy in CLL treatment [6, 7], while in AML, combined treatment with other drugs was found to be promising [3]. Recently, Venetoclax in combination with hypomethylating agents (HMA) Azacytidine or Decitabine has been approved for the first‐line therapy of elderly (> 65 years) patients and/or those with comorbidities, unable to undergo the 3 + 7 regimen [8]. Triplet combinations of Venetoclax + Azacytidine with a drug targeting a specific mutation of the particular patient are used [9, 10]. However, Venetoclax treatment often leads to adaptive resistance [11], which is likely governed mainly by the upregulation of another antiapoptotic protein, Mcl‐1 [12]. Other recently published resistance mechanisms involve metabolic adaptation [13] or aberrant methylation of CpG islands within the BBC3 promoter, mediating Puma downregulation at both the transcript and protein level [14].

The role of reactive oxygen species (ROS) in AML has been widely discussed [15, 16, 17, 18] and ROS induction is known to occur also during the Venetoclax treatment [19]. It likely associates with Bcl‐2 inhibition, as Bcl‐2 operates at mitochondria and any disequilibrium of Bcl‐2‐family members may lead to aberrant ROS production. Bcl‐2 phosphorylation at Serine 70 (S70) has been reported to act as a redox sensor, preventing excessive ROS production at mitochondria, thereby protecting DNA and cell survival [20]. Venetoclax‐mediated Bcl‐2 inhibition decreases its activity and is manifested by the S70 dephosphorylation [21] and hence by the ROS increase. Moreover, in combination regimen, Venetoclax was found to disrupt the HMA (specifically Decitabine)‐induced antioxidant defense system mediated by Nrf2 activation [22]. Bcl‐2 downregulation and ROS production are widely reported also after treatment with Doxorubicin, an anthracycline formerly used in 3 + 7 IC regimen [23, 24, 25]. Doxorubicin induces extensive DNA damage causing nucleolar stress and p53‐dependent apoptosis [26, 27]. During this process, nucleolar defragmentation occurs together with a release of nucleolar proteins into the nucleoplasm.

Nucleolin (NCL) is the most abundant nonribosomal nucleolar phosphoprotein and its redistribution in response to nucleolar stress is widely documented [28, 29]. NCL is essential for normal nucleolar function, playing important roles in ribogenesis, cell cycle regulation, DNA‐damage repair, and apoptosis [30]. Major phosphorylation sites of NCL are Serines and Threonines [31]. Serine phosphorylation is mediated by CKII and related to nucleolar function in the control of rDNA transcription [32], while Threonine is phosphorylated by cdk1 kinase during mitosis [33, 34]. Sequential CKII and cdk1 phosphorylation modulates NCL function in regulating nucleolar structure and activities between interphase and the mitotic phase during cell cycle progression [31]. NCL is often upregulated in cancer [35, 36] and its overexpression stabilizes Bcl‐2 mRNA, which contributes to apoptosis inhibition [37, 38, 39]. Interaction of NCL with another nucleolar phosphoprotein nucleophosmin (NPM) is abrogated by AML‐characteristic NPM mutation [40, 41]. High NCL expression on the cell surface is an indicator of cancer progression [42]. Targeting cancer‐related NCL aberrations thus shows promising potential for new therapeutic strategies.

Here we report internal heterogeneity of a cell culture exposed to Venetoclax, manifested by generation of two distinct subpopulations with markedly different response. These subpopulations differ in ROS content and mitochondrial activity. In addition, the Venetoclax‐sensitive cells showed significant decrease in NCL protein level and phosphorylation. Monitoring of ROS with different fluorescence probes revealed negative correlation between two ROS indicators, suggesting participation of multiple ROS types during Venetoclax action. For comparison, Bcl‐2 inhibition, apoptosis markers, ROS production, and NCL changes after Doxorubicin, Decitabine, and their combinations with Venetoclax are presented. The effect of subtoxic Venetoclax concentration on all these parameters during 96 h treatment indicates that after sensitive subpopulation clearance, the remaining cells proliferate, eventually recovering the culture.

## 2 Material and Methods

### 2.1 Cell Culture and Chemicals

AML cell lines OCI‐AML2, OCI‐AML3, MV4‐11, and MOLM‐13 were purchased from DSMZ (Braunschweig, Germany). Mantle cell lymphoma cell line HBL‐2 was kindly provided by prof. Klener (Institute of Pathological Physiology, Charles University, Prague). OCI‐AML2 and OCI‐AML3 were cultivated at 37°C and 5% CO_2_ in α‐MEM medium with 20% FBS (Biosera, Cholet, France), antibiotics (100 U/mL penicillin, 100 μg/mL streptomycin; Merck, Darmstadt, Germany), and 1% L‐glutamine (Merck). MV4‐11, MOLM‐13, and HBL‐2 were cultivated in RPMI‐1640 medium (Biosera) with 10% FBS and antibiotics and L‐glutamine as described above. All the cell cultures were regularly tested for mycoplasma.

Primary cells of AML patients with hyperleukocytosis were obtained by leukapheresis at diagnosis, before therapy initiation. Detailed information on primary AML samples is provided in Table S1. All patients signed informed consent to the use of their biological material for research purposes. The leukapheretic products were diluted 20‐fold in phosphate buffered saline (PBS) and the mononuclear cell fraction was separated using Histopaque‐1077 (Merck). Primary cells were cultivated in RPMI‐1640 medium with 10% FBS and antibiotics and L‐glutamine.

Venetoclax, Doxorubicin, and Decitabine were purchased from Selleckchem (Planegg, Germany) and added to cell suspension from 5 to 10 mM stock solution to final concentration 0.5–1 μM, as specified in text. 6‐Carboxy‐2′,7′‐dichlorodihydrofluorescein diacetate (H_2_DCFDA, Thermo Fisher Scientific, Waltham, MA, USA #C400), CellROX Green, Orange and DeepRed probes (Thermo Fisher Scientific, #C10448), JC‐1 (#HY‐15534, MedChemExpress), and propidium iodide (PI, Merck, #81845) were diluted to the working concentrations from 5 mM stock solutions. Stock solution of Tetramethylrhodamine Methyl Ester (TMRM, Invitrogen, #T668) was 1 mM. PE‐labeled Annexin V (#556421) was purchased from BD Pharmingen. N‐acetyl‐l‐cysteine (Merck, #A9165) was diluted from 200 mM stock solution.

### 2.2 Cell Viability and Proliferation

Cell viability and proliferation were assessed by the Trypan Blue exclusion method using BioRad Cell Counter (BioRad, Hercules, CA, USA). At each time point of treatment, the fraction of live cells and their concentration relative to control (untreated) sample were evaluated.

### 2.3 Immunoblotting

The cells were washed with PBS and lysed in Laemmli sample buffer (50 mM Tris pH 6.8, 2% SDS, 100 mM DTT, 10% glycerol), boiled at 95°C for 5 min, centrifuged at 200 000*g* at 4°C for 4 h and the supernatant was stored at −20°C. Five to 10 μL of each sample were loaded onto SDS–PAGE gels and transferred into PVDF membrane (BioRad). Mouse monoclonal antibodies against β‐Actin (sc‐47 778), Bcl‐2 (sc‐509), caspase‐3 (sc‐7272), Mcl‐1 (sc‐12756), NPM (sc‐70392), PARP (sc‐8007), p21 (sc‐6246), p53 (sc‐126), and PUMA (sc‐374223) were purchased from Santa Cruz Biotechnology and were used at a dilution 1:250–1:500. Rabbit monoclonal antibodies against c‐Myc (ab32072), CKIIα (ab76040), and phospho‐CKII (ab119410, all Abcam, UK) were used diluted 1:500. Rabbit monoclonal antibodies against NCL (ab129200), NCLpT76 (ab168363), and NCLpT84 (ab155977) from Abcam were used at 1:1000 dilution. Polyclonal rabbit antibody for detection of Bcl‐2 phosphorylated at Ser70 (#2827S), and monoclonal rabbit antibodies from Cell Signaling Technology (Danvers, MA, USA) against cdk1 (#77055S), cdk2 (#2546S), cdk‐phosphoY15 (#9111S), and cdk‐phosphoT160/161 (#2561S) were diluted 500×. Antimouse and antirabbit HRP‐conjugated secondary antibodies were purchased from Thermo Fisher Scientific and used at concentrations 1:10000–1:50000. ECL Plus Western Blotting Detection System (GE Healthcare, Chicago, IL, USA) or SuperSignal West Atto Ultimate Sensitivity Substrate (Thermo Fisher Scientific) were used for chemiluminescence detection and signals were detected by G‐box Chemi XX6 digital imaging device (Syngene, Cambridge, UK).

### 2.4 Flow Cytometry

#### 2.4.1 Cell Cycle

For cell cycle measurement, treated cells were collected in PBS, fixed in 70% EtOH, and stored at −20°C until used. After extensive washes with ice‐cold PBS, the cells were resuspended in PI‐staining solution (50 μg/mL propidium iodide (PI), 100 μg/mL RNase A, 0.1% (v/v) Triton X100 in PBS), incubated for 2 h in the dark at 4°C, and measured by flow cytometer BD Fortessa (BD Life Sciences, Franklin Lakes, NJ, USA). Cell cycle distribution was evaluated using FlowJo Software (BD Life Sciences).

#### 2.4.2 ROS Content and Mitochondrial Membrane Potential

Treated cells were washed with PBS and incubated with 5 μM H_2_DCFDA for 30 min/37°C. After H_2_DCFDA removal, the cells were resuspended in 1 μg/mL PI/PBS and immediately measured by flow cytometer. Mean fluorescence intensity (MFI) was evaluated as a difference between H_2_DCFDA‐stained and unstained cells, and the H_2_DCFDA‐positive fraction of live (PI‐negative) cells was evaluated using FlowJo. For CellROX reagents, TMRM and JC‐1 staining, the probes were added directly to the cell culture to final concentrations of 5 μM, 100 nM, and 2 μM, respectively, and incubated at 37°C for 30 min (CellROX) or 1 h (TMRM, JC‐1). JC‐1 signal was evaluated as the ratio of intensities detected with ex561/em586/15 (orange fluorescence from aggregates) and ex488/em530/30 (green signal from monomers). For combined staining, the CellROX Deep Red and TMRM‐labeled cells were washed with PBS and subsequently stained with H_2_DCFDA.

#### 2.4.3 Annexin V Staining

PE Annexin V was used according to manufacturer's instructions. Briefly, cells were washed with PBS, resuspended in 100 μL of annexin‐binding buffer (ABB: 10 mM Hepes, 140 mM NaCl, 2.25 mM CaCl_2_, pH 7.4) with 5 μL/100 μL of PE_Annexin V and left at RT in dark for 15 min. Then 400 μL of ABB was added and the sample was immediately analyzed by flow cytometer. For combined staining, incubation with CellROX Deep Red and H_2_DCFDA labeling preceded the PE Annexin V protocol.

#### 2.4.4 Cell Sorting

Treated cells were stained with H_2_DCFDA and PI as in Section 2.4.2 and sorted at BD FACS Discover S8 (BD Life Sciences). H_2_DCFDA‐low/PI‐negative and H_2_DCFDA‐high/PI‐negative populations were further examined by immunoblot (Section 2.3). Alternatively, cell cycle distribution in individual populations was tested (Section 2.4.1).

#### 2.4.5 Intracellular Staining

Treated cells were collected in PBS and fixed in 4% PFA for 15 min. Then they were fixed either in ice‐cold methanol for 30 min or with 0.5% Triton X‐100 for 10 min/RT. After extensive washing and blocking with 1% BSA for 20 min, primary anti‐NCL/anti‐NCLpT84 and AlexaFluor‐conjugated secondary antibodies were sequentially applied similarly as for microscopy (Section 2.5).

### 2.5 Fixation and Immunostaining for Microscopy

For immunofluorescence staining, the cells in PBS were seeded onto coverslip in wet chamber for 30 min/RT, gently dried and fixed with 4% PFA overnight/4°C. After permeabilization with 0.2% Triton X‐100 (10 min/RT), primary antibodies, mouse anti‐NCL (sc‐17826, Santa Cruz Biotechnology, 1:50) and rabbit anti‐NCLp76 (ab168363, Abcam, 1:100) were added for 1 h/RT. After three times washing with PBS/0.2% Tween20, the cells were incubated (1 h/RT) with antimouse AlexaFluor488 and antirabbit AlexaFluor555‐conjugated secondary antibodies (Thermo Fisher) and Hoechst33342 (Merck). After staining, the coverslips were mounted to microscopic slide using Prolong (Thermo Fisher) and observed under confocal fluorescence microscope (Olympus FV1000) with 60× oil immersion objective (NA1.35).

### 2.6 qPCR

Total RNA was isolated from 2 × 10^7^ cells using RNeasy Mini kit (Qiagen #74104) and reverse transcribed to cDNA with SensiFAST cDNA Synthesis Kit (Bioline, London, UK, #BIO65054). Relative amounts of BCL2, MCL1, NCL, and BBC3 transcript were calculated from the expression ratio of particular gene and GAPDH qPCR products measured using the SensiFAST SYBR No‐ROX Kit (Bioline #BIO98020). The respective forward and reverse primer sequences were: GGTGGAGGAGCTCTTCAGG and ACAGTTCCACAAAGGCATCC for BCL2, TAAGGACAAAACGGGACTGG and ACCAGCTCCTACTCCAGCAA for MCL1, GAGCTCTCGCTGGCCTTCGG and TGAAGCGGACAAGTGGCGCA for NCL, CTCTCCTCTCGGTGCTCCTT and AGGCTAGTGGTCACGTTTGG for BBC3, and GAAACTGTGGCGTGATGGC and CCGTTCAGCTCAGGGATGAC for GAPDH.

### 2.7 Statistical Evaluation

The intensity of ECL signal was evaluated using GeneTools Software (Syngene). The intensity of bands from immunoblots was related to the value of the control sample and standardized to the β‐Actin level. GraphPad Prism software (version 7.03, GraphPad Software, San Diego, California USA) was used for statistical evaluation of experimental data. Unpaired *t*‐test or ANOVA tests with multiple comparisons have been performed as specified in figure captions.

## 3 Results

### 3.1 Chemotherapy‐Induced Proliferation Arrest, Loss of Viability, and Cell Cycle Distribution Changes

**FIGURE 1:**
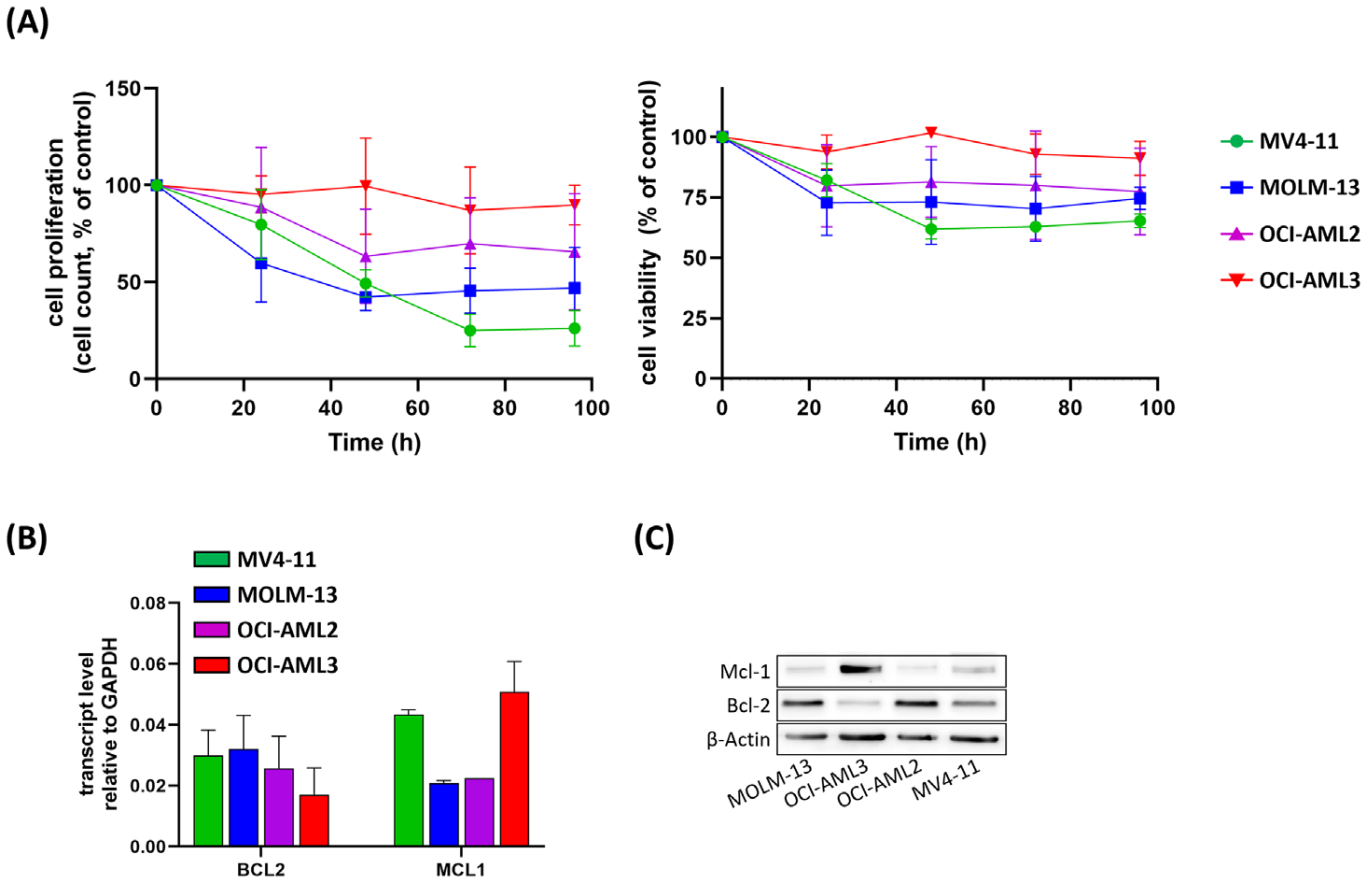
(A) Effect of 0.5 μM Venetoclax on AML cell lines MV4‐11 (green circles), MOLM‐13 (blue squares), OCI‐AML2 (violet triangles), and OCI‐AML3 (red inverted triangles): Time course of proliferation (left) and viability (right) relative to untreated control. (B) mRNA level of BCL2 and MCL1 and (C) protein level of Bcl‐2 and Mcl‐1 in individual cell lines. Mean values are plotted with ±SD.

We tested the effect of Venetoclax on proliferation and viability of AML cells (Figure 1). From a panel of four AML cell lines, one line (OCI‐AML3) was resistant while three lines (MV4‐11, MOLM‐13, OCI‐AML2) exhibited varying degrees of sensitivity (MV4‐11 > MOLM‐13 > OCI‐AML2) to 0.5 μM Venetoclax. We therefore analyzed Bcl‐2 mRNA and protein expression in all tested cell lines (Figure 1B,C), and consistently with viability results, we found lower Bcl‐2 expression in OCI‐AML3 cell line compared to the others. In addition, high expression of Mcl‐1, which is known to alternate some Bcl‐2 functions, has been detected in the resistant OCI‐AML3 line. To compare the Venetoclax with other leukemia therapy‐related drugs, we monitored the proliferation and viability of cells treated with 1 μM Doxorubicin or 1 μM Decitabine (Figure S1). All cell lines were sensitive to Doxorubicin and the addition of Venetoclax did not alter this response. Decitabine caused growth arrest and slight viability decrease in MV4‐11 and MOLM‐13 while OCI‐AML2 and OCI‐AML3 remained unaffected. Impact of combination of Decitabine and Venetoclax was mostly less than additive.

**FIGURE 2:**
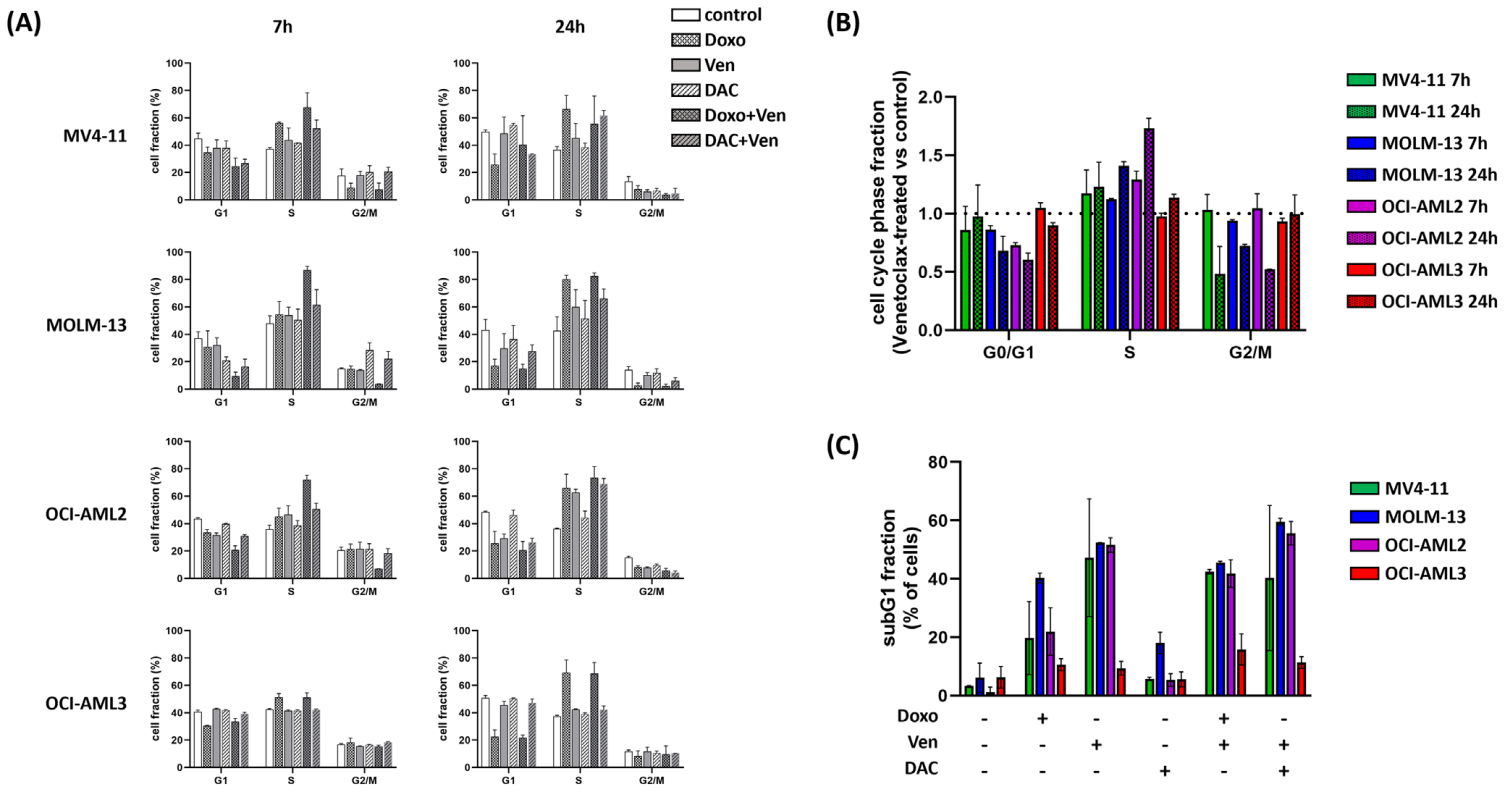
(A) Effect of 1 μM Doxorubicin (Doxo), 0.5 μM Venetoclax (Ven), 1 μM Decitabine (DAC) and their combinations on cell cycle distribution after 7 h (left) and 24 h (right) treatment in individual AML cell lines. (B) Distribution of cell cycle phases after 7 h (clear bars) and 24 h (dotted bars) treatment with 0.5 μM Venetoclax relative to control (untreated) sample and (C) a fraction of cells in subG1 phase at 24 h‐point in AML cell lines MV4‐11 (green), MOLM‐13 (blue), OCI‐AML2 (violet), and OCI‐AML3 (red). Mean values are plotted with ±SD.

Drug‐induced proliferation and viability changes closely associate with alterations in cell cycle distribution (Figure 2A,B). Doxorubicin caused significant enrichment of S‐phase fraction followed by loss of viability manifested by subG1 fraction enrichment (Figure 2C). Decitabine induced transient G2/M‐arrest in the sensitive cells, which diminished after 24 h. This corresponds to the proliferation arrest but no effect on viability documented in Figure S1. Venetoclax caused stepwise accrual of S‐phase fraction and G2/M drop after 24 h‐treatment in the sensitive cells, and almost no change in the resistant cells (Figure 2A,B). Venetoclax combinations with either Doxorubicin or Decitabine mainly reflected changes induced by these drugs alone.

### 3.2 Bcl‐2 Inhibition and Apoptosis Induction

**FIGURE 3:**
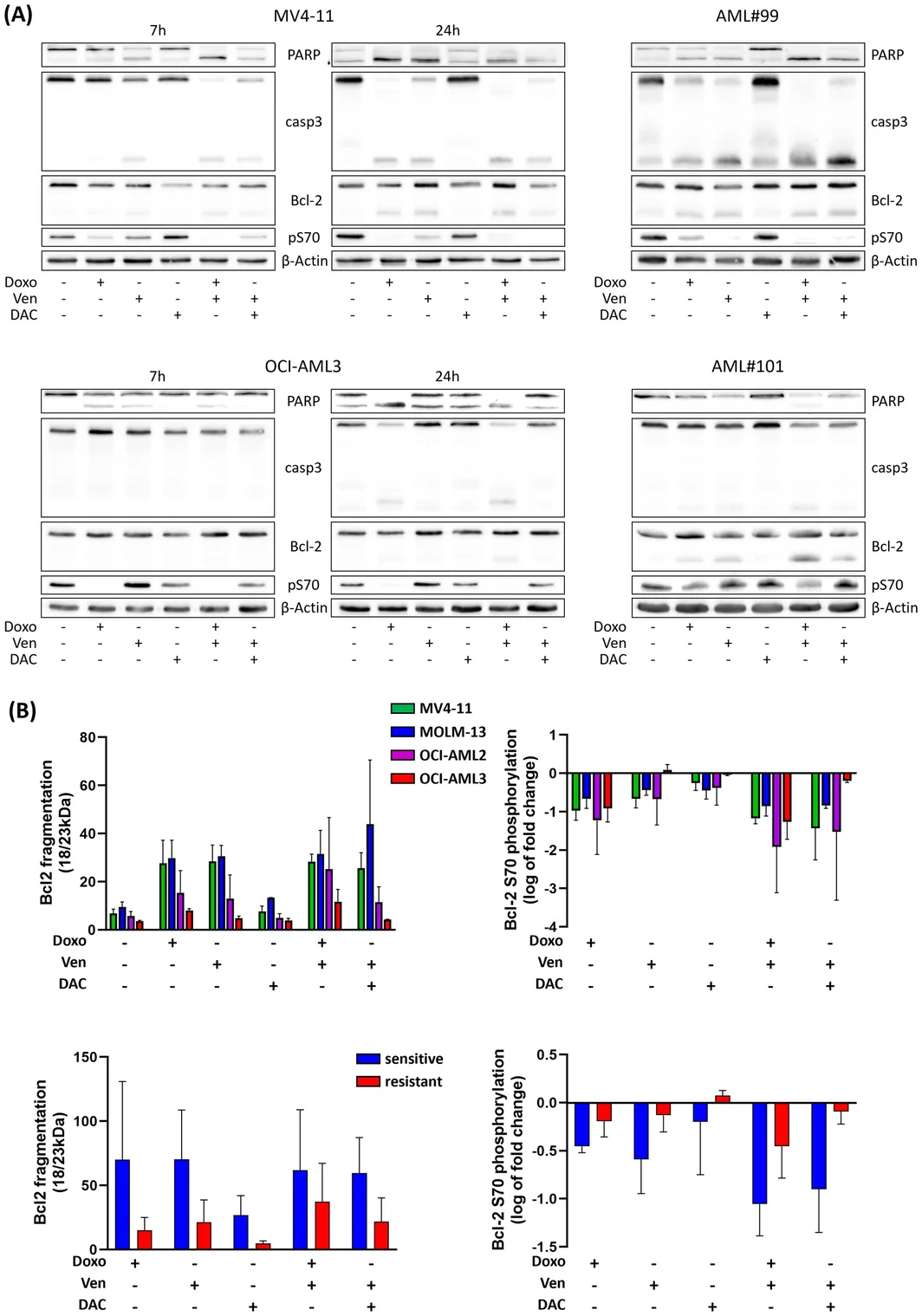
Effect of 7 h (cell lines) and 24 h (all samples) treatment with 1 μM Doxorubicin (Doxo), 0.5 μM Venetoclax (Ven), 1 μM Decitabine (DAC), or their combinations on AML cells. (A) Representative images of PARP fragmentation, caspase‐3 activation, Bcl‐2 fragmentation, and phosphorylation of Bcl‐2 at Serine70 (pS70) in Venetoclax‐sensitive (upper panel) and Venetoclax‐resistant (lower panel) samples. β‐Actin serves as a loading control. (B) Statistical evaluation of Bcl‐2 fragmentation and phosphorylation in cell lines (upper panel) and patient samples (lower panel). For Bcl‐2 phosphorylation, values are presented as log of change relative to control (untreated) sample. Mean values are plotted with ±SD.

Protein markers of apoptosis in samples treated with Doxorubicin/Venetoclax/Decitabine or combinations were analyzed by immunoblot. Besides PARP cleavage and caspase‐3 activation, apoptosis‐related fragmentation and dephosphorylation of Bcl‐2 were investigated in samples from our AML lines panel as well as from primary cells of AML patients (Figure 3). Consistently with proliferation and viability results, increased apoptotic parameters were detected in all Doxorubicin‐treated samples and in the three cell lines displaying viability loss after Venetoclax. Similarly, we classified our primary AML samples according to their response to Venetoclax: significant PARP and caspase‐3 cleavage identified sensitive samples while the others were categorized as resistant. As seen from Figure 3B, sensitivity to Venetoclax predicts altered Bcl‐2 responses to other drugs, suggesting shared pathways affected by these drugs.

**FIGURE 4:**
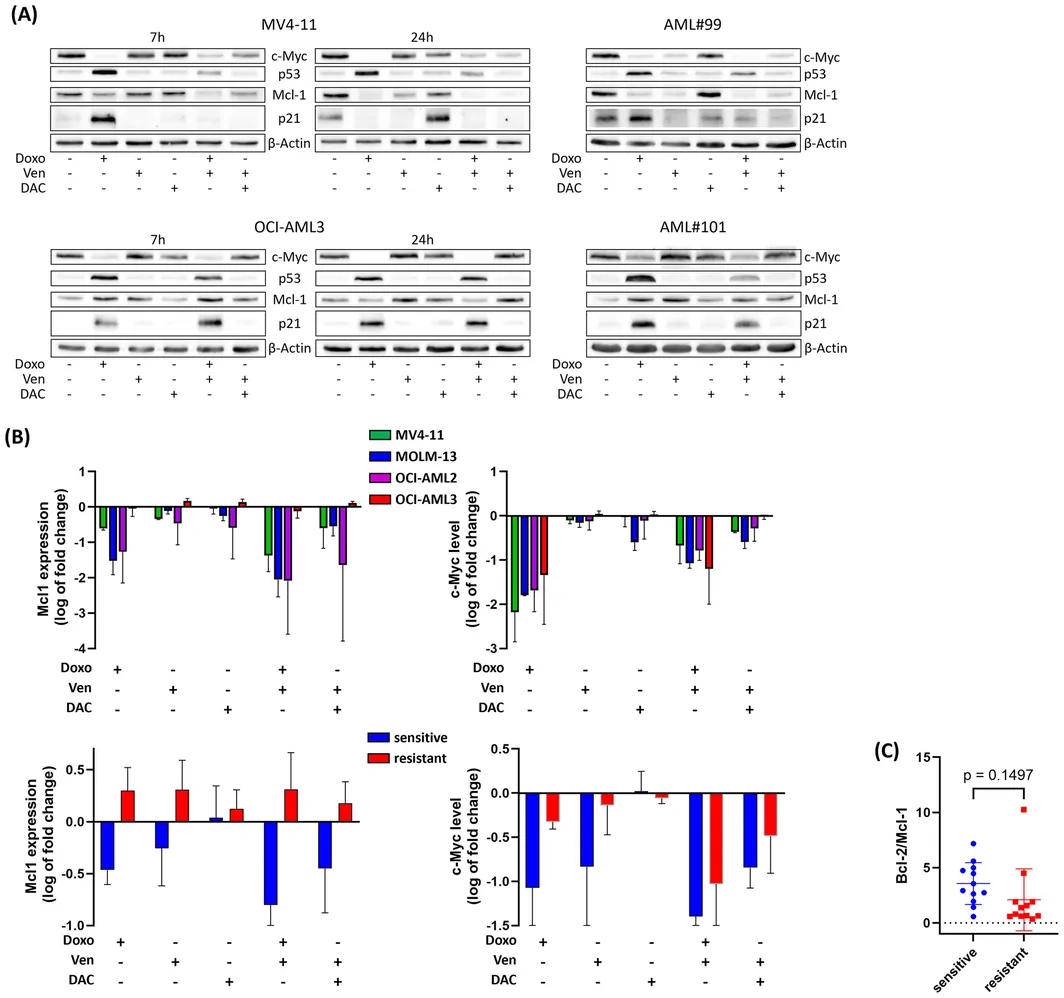
Effect of 7 h (cell lines) and 24 h (all samples) treatment with 1 μM Doxorubicin (Doxo), 0.5 μM Venetoclax (Ven), 1 μM Decitabine (DAC) or their combinations on AML cells. (A) Representative images of Mcl‐1 and c‐Myc protein levels in Venetoclax‐sensitive (upper row) and Venetoclax‐resistant (lower row) samples. β–Actin serves as a loading control. (B) Statistical evaluation of Mcl‐1 and c‐Myc protein levels in cell lines (upper row) and patient samples (lower row). Data are presented as log of change relative to control (untreated) sample. (C) Ratio of Bcl‐2 and Mcl‐1 protein levels in AML patient samples. Mean values from biological replicates are plotted with ±SD. Unpaired *t*‐test was used for *p*‐value calculation.

Among other apoptosis‐related proteins, decreased Mcl‐1 and c‐Myc levels were detected in Venetoclax‐sensitive cells (Figure 4A,B) while the resistant cells responded by Mcl‐1 upregulation. This is again consistent with the known fact that Mcl‐1 can bridge the Bcl‐2 inhibition. Similarly to the results from cell lines, the basal Bcl‐2/Mcl‐1 ratio is higher in Venetoclax‐sensitive compared to the resistant samples of primary cells (Figure 4C). Interestingly, Doxorubicin‐induced p53 and p21 increases were markedly attenuated by Venetoclax addition in sensitive cells. Furthermore, p21 elevation after Doxorubicin was only transient and diminished in 24 h‐treated sensitive samples. Contrary to this, p53‐independent p21 induction occurred after 24 h Decitabine treatment in sensitive but not resistant cells.

### 3.3 Venetoclax Causes Nucleolin Alterations

**FIGURE 5:**
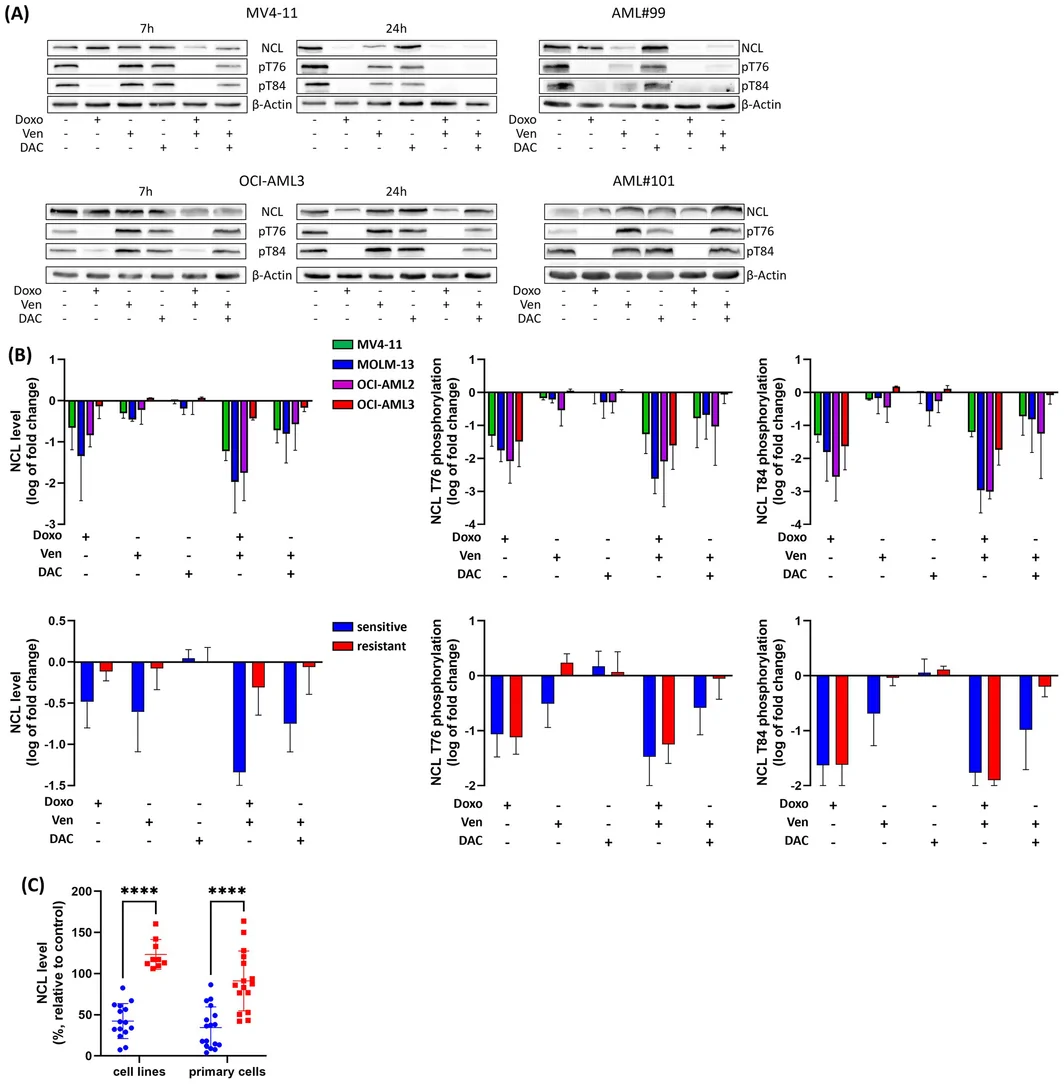
Effect of 7 h (cell lines) and 24 h (all samples) treatment with 1 μM Doxorubicin (Doxo), 0.5 μM Venetoclax (Ven), 1 μM Decitabine (DAC), or their combinations on AML cells. (A) Representative images of NCL protein level and specific phosphorylations at Threonines 76 and 84 (pT76 and pT84) in Venetoclax‐sensitive (upper panel) and Venetoclax‐resistant (lower panel) samples. β‐Actin serves as a loading control. (B) Statistical evaluation of NCL protein level and phosphorylations in cell lines (upper panel) and patient samples (lower panel). Data are presented as log of change relative to control (untreated) sample. (C) Comparison of NCL protein level in treated cells relative to control in AML cell lines and patient samples. Mean values from biological replicates are plotted with ±SD. Unpaired *t*‐test was used for *p*‐value calculation (****p < 0.0001).

**FIGURE 6:**
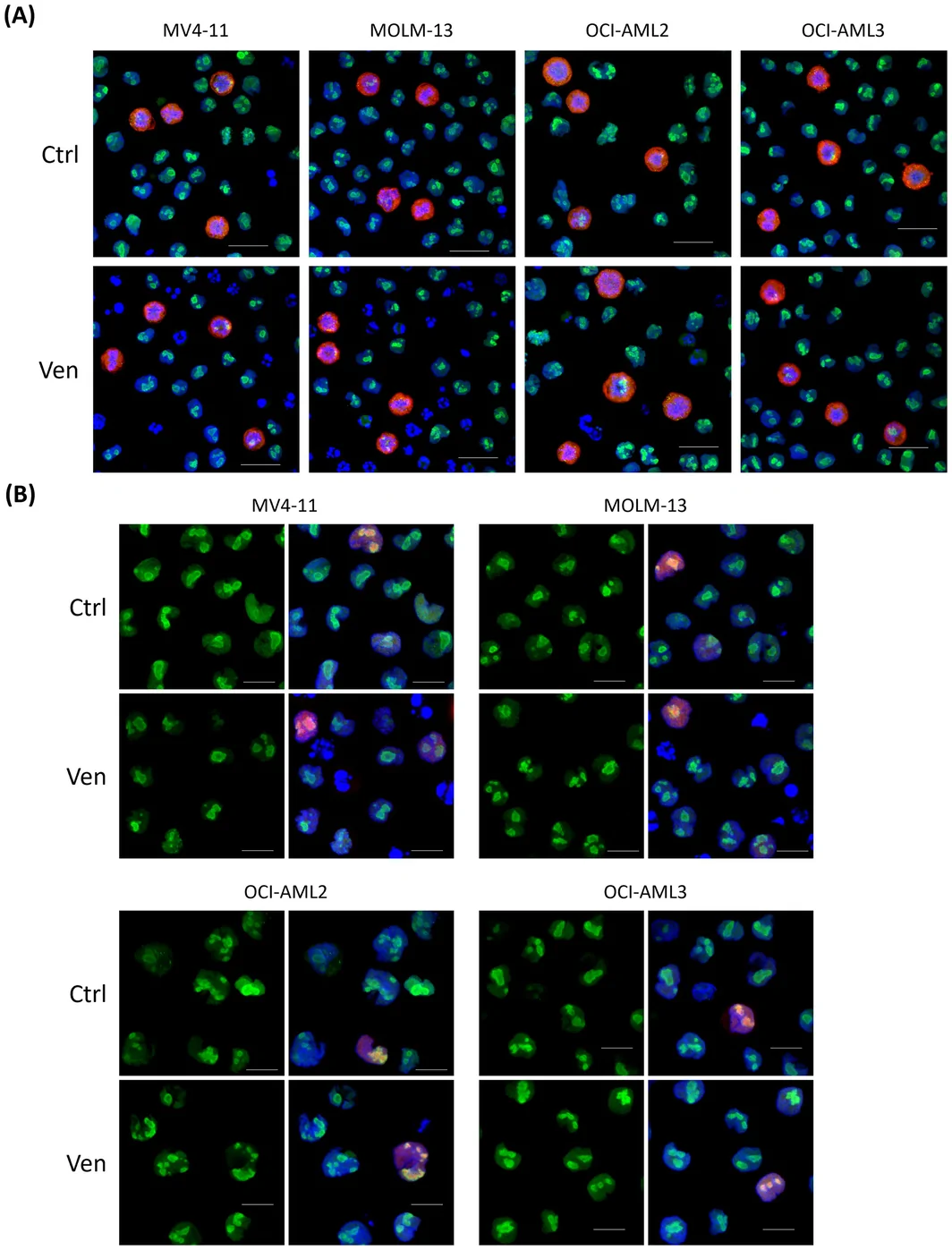
Effect of 20 h Venetoclax treatment on NCL distribution in AML cell lines. Fixed and permeabilized control (Ctrl) and 0.5 μM Venetoclax‐treated cells (Ven) were stained for total NCL (AlexaFluor488, green) and NCL phosphorylated at T76 (AlexaFluor555, red). Nuclei were counterstained with Hoechst33342. Bar represents 20 μm (A) or 10 μm (B).

**FIGURE 7:**
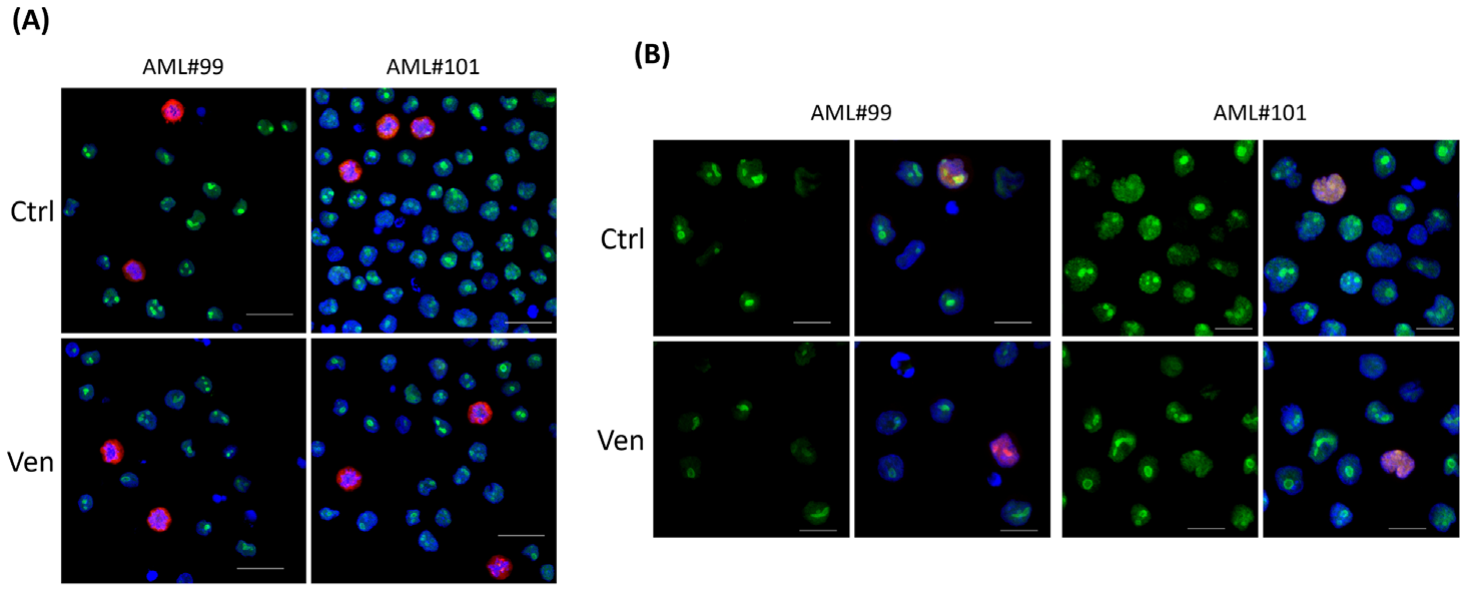
Effect of 0.5 μM Venetoclax on NCL distribution in AML patient samples: AML#99 represents sensitive cells, AML#101 patient sample was resistant to Venetoclax. Fixed and permeabilized control (Ctrl) and 0.5 μM Venetoclax‐treated cells (Ven) were stained for total NCL (AlexaFluor488, green) and NCL phosphorylated at T76 (AlexaFluor555, red). Nuclei were counterstained with Hoechst33342. Bar represents 20 μm (A) or 10 μm (B).

Nucleolar protein nucleolin (NCL) is known to be overexpressed in cancers and its role in apoptosis is widely described. We investigated NCL behavior in our samples treated with Venetoclax, Doxorubicin, Decitabine, and their combinations (Figure 5A,B). Venetoclax treatment caused a significant decrease in NCL protein level as well as dephosphorylation of NCL. In particular, Threonines at positions 76 and 84 have been dephosphorylated in all sensitive samples but not in the resistant ones. This difference was highly significant for both AML cell lines and primary AML samples (Figure 5C). Rapid and marked NCL dephosphorylation was also detected after Doxorubicin treatment. However, total NCL protein level remained unchanged during several hours after treatment. Doxorubicin‐caused NCL dephosphorylation is thus likely due to cell cycle arrest and dramatic decrease of mitotic cell percentage. Conversely, confocal microscopy (Figure 6) revealed a comparable fraction of mitotic cells (characterized by high intensity of NCLpT76 signal) in control and Venetoclax‐treated samples. Detailed analysis of NCL subcellular localization indicates an increased ratio of nucleolar vs. nucleoplasmic NCL after Venetoclax treatment in the most sensitive cells (MV4‐11, MOLM‐13, Figure 6B), suggesting the disappearance of NCL from the nucleoplasm by Venetoclax. Similar results were obtained also with primary cells (Figure 7), although the subcellular localization was hard to evaluate due to low sample concentration.

Level and activity of main kinases regulating NCL phosphorylation, casein kinase II (CKII) and cyclin‐dependent kinases cdk1 and cdk2 were also investigated (Figure S2). Inhibiting phosphorylation of cdks at Tyrosine 15 (denoted Y15) decreased after both Doxorubicin and Venetoclax treatment in sensitive cells, while activating phosphorylation at Threonine 160/161 (denoted T161) slightly increased. The CKII phosphorylation was barely detectable and showed no significant changes compared to control. These results rather suggest increased activity of the cdk kinases regulating the NCL phosphorylation. However, the cdks' activity depends on their interaction with various cyclins and is affected by other molecules entering the cdk‐cyclin complex. In the Doxorubicin‐treated cells, the p53‐induction of p21 probably causes the cdk inhibition and subsequent NCL dephosphorylation. On the other hand, the changes induced by Venetoclax are milder and may associate with decrease of total NCL level.

To further extend our findings on Venetoclax effect on NCL, we also performed RNA analysis of Venetoclax‐treated cells (Figure S3). However, we detected only insignificant downregulation of BCL2 transcript and no change in NCL mRNA level. Among several other apoptosis‐related genes, the BBC3 gene was significantly upregulated by Venetoclax in sensitive cells. However, the level of Puma, a protein coded with the BBC3 gene, decreased regardless of Venetoclax sensitivity (Figure S3B).

### 3.4 Drug‐Induced Oxidative Stress

**FIGURE 8:**
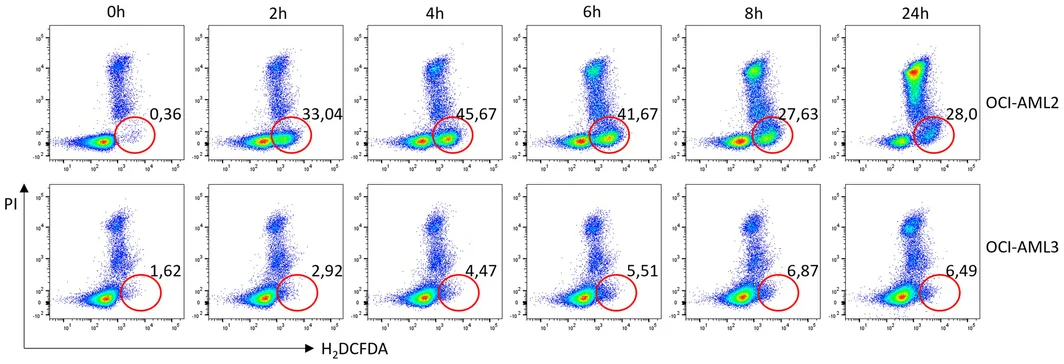
Representative flow‐cytometry image of time evolution of ROS production in samples treated with 0.5 μM Venetoclax (Ven). Fraction of H_2_DCFDA‐positive cells is marked by red circle and the numbers denote frequency of ROS‐positive subpopulation in the live (PI‐negative) cell population. Results from sensitive OCI‐AML2 cell line vs resistant OCI‐AML3 cells are presented.

**FIGURE 9:**
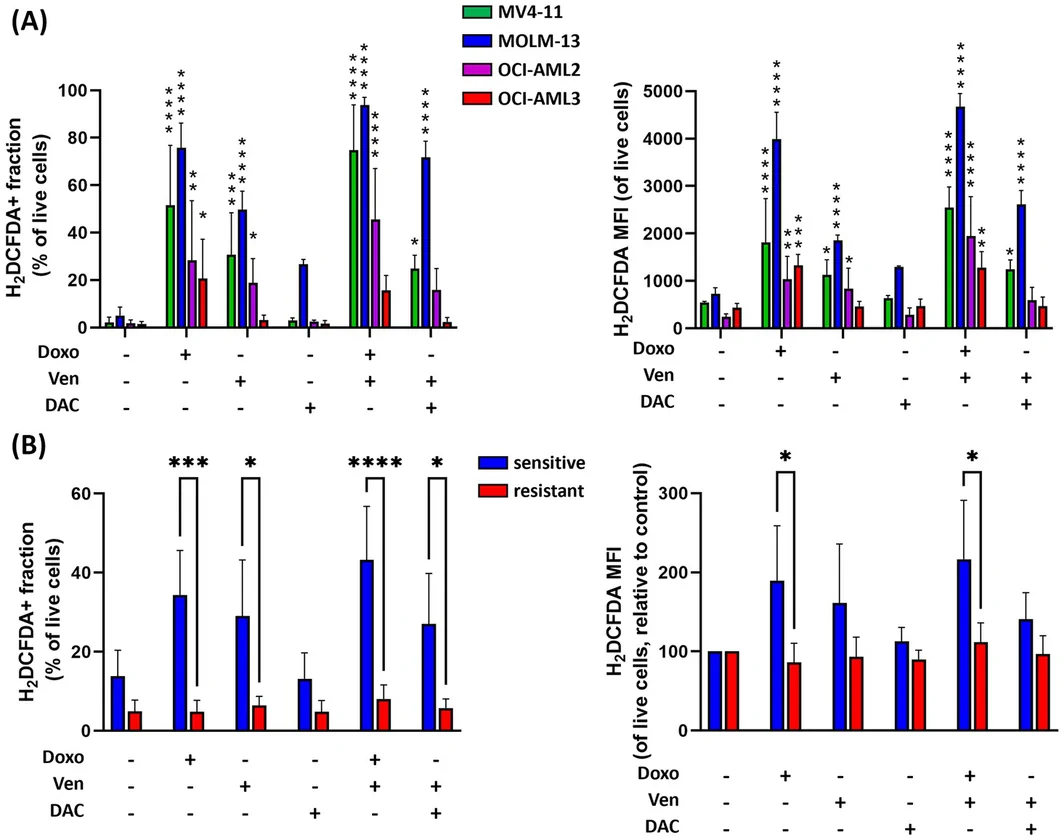
ROS production in AML cell lines (A, upper row) and patient samples (B, lower row) treated for 24 h with 1 μM Doxorubicin (Doxo), 0.5 μM Venetoclax (Ven), 1 μM Decitabine (DAC) or their combinations. Fraction of ROS‐positive cells (left) and mean of H_2_DCFDA fluorescence intensity relative to control (MFI, right) were evaluated. Mean values are plotted with ±SD. Asterisks mark statistical significance of difference against appropriate control sample (A) or of a difference between sensitive and resistant samples (B). Data were processed by Two‐way ANOVA with multiple comparisons and *p*‐values calculated from at least three repeated experiments. *p < 0.05, **p < 0.01, ***p < 0.001, ****p < 0.0001.

Venetoclax has been reported to induce ROS generation. We therefore tested ROS levels in our samples by flow‐cytometry, first using H_2_DCFDA staining. We detected increased H_2_DCFDA fluorescence intensity in Venetoclax‐treated samples, but surprisingly this change did not manifest as a simple shift of fluorescence peak. Instead, it consisted of a slight shift of basal peak and occurrence of a second peak with higher fluorescence intensity. Simultaneous staining with propidium iodide (PI) revealed a PI‐negative/H_2_DCFDA‐positive population in the sensitive cell line while this population was negligible in the resistant OCI‐AML3 cell line (Figure 8). The H_2_DCFDA‐positive fraction increased during the first hours of drug exposure and eventually shifted to PI‐positivity. The time course of MFI increase and growth of the H_2_DCFDA‐positive subpopulation were comparable (Figure S4), suggesting that the positive fraction can be used as a parameter independent of the initial intensity, instead of MFI, for ROS production evaluation. Similar results were obtained with primary AML cells, although due to the low number of patient samples the MFI changes did not reach statistical significance (Figure 9). Interestingly, the positive subpopulation was also detected in Doxorubicin‐treated cells; however, this subpopulation occurred as late as after 24 h drug treatment (Figure S4).

**FIGURE 10:**
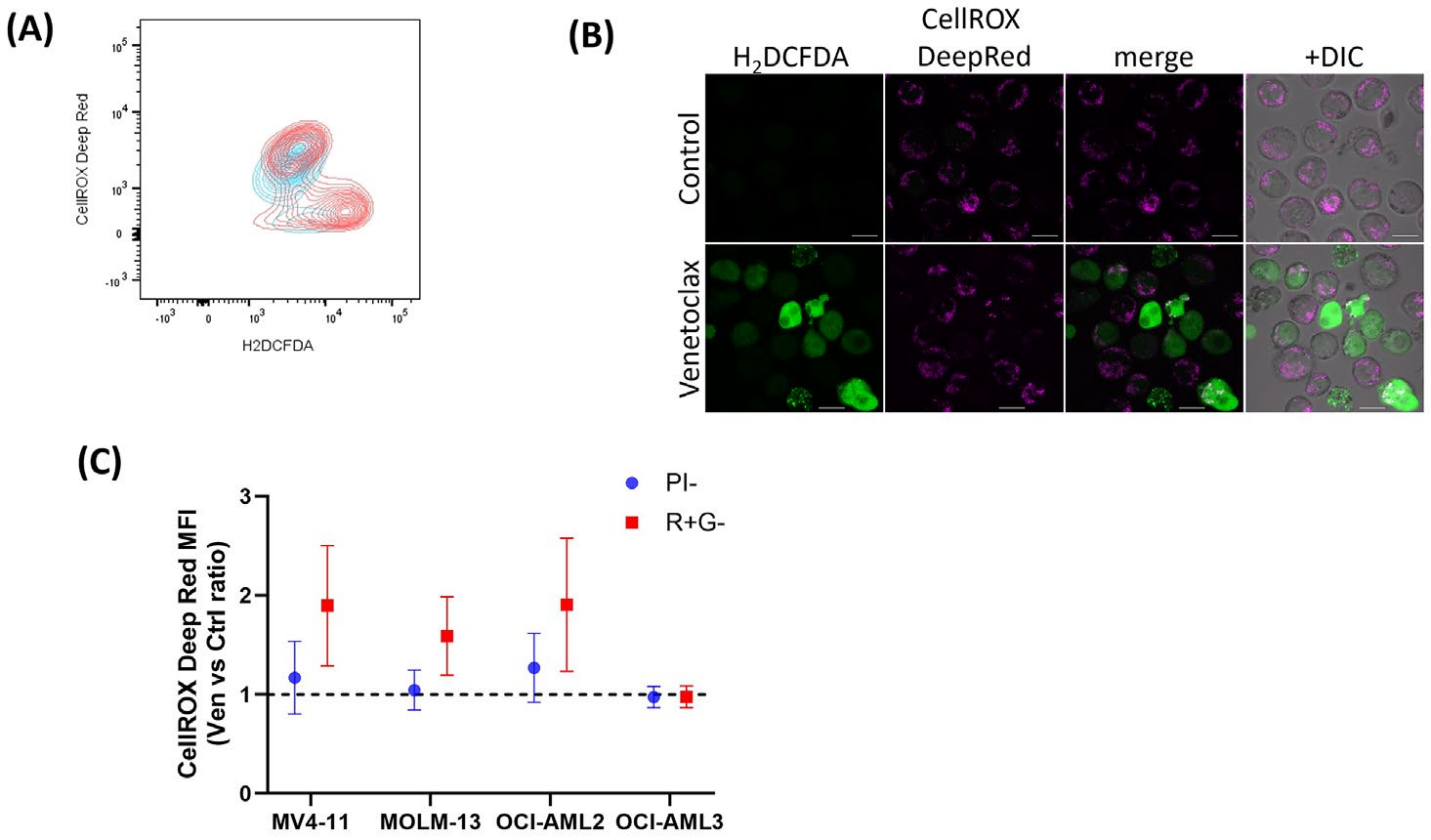
Co‐staining with CellROX Deep Red and H_2_DCFDA, untreated (cyan) and 0.5 μM Venetoclax‐treated (red) MOLM‐13 cells. (A) Representative graph shows negative correlation between the two probes. Increased CellROX Deep Red signal from the “resistant” subpopulation and enhanced H_2_DCFDA intensity from sensitive cells are visible. (B) Microscopy images document different localization and intensities of the CellROX Deep Red (magenta) and H_2_DCFDA (green) in individual cells. (C) CellROX Deep Red MFI from PI‐negative cells (PI−, blue) and the CellROX Deep Red‐high/H_2_DCFDA‐low subpopulation (*R*+G−, red) relative to control group in AML cell lines (dotted line at ratio value = 1). Mean ± SD values from at least four experiments are plotted.

To better characterize subpopulations induced by Venetoclax treatment, we examined ROS induction using CellROX probes (Figure S5). All three probes consistently showed two subpopulations induced by Venetoclax, one with lower signal and the other with higher MFI compared to untreated sample (Figure S5A). This resulted in high variations in MFI from whole sample, depending on size of the Venetoclax‐sensitive fraction (Figure S5B), but the signal from the CellROX‐positive fraction of Venetoclax‐treated sample was consistently enhanced (Figure S5C). CellROX Deep Red variant was selected for subsequent experiments owing to its spectral compatibility with H_2_DCFDA and propidium iodide staining. Surprisingly, we found a negative correlation between H_2_DCFDA and CellROX Deep Red signal in co‐stained cells (Figure 10). In addition, microscopy images show different localization of the two probes: while relatively homogeneous intracellular signal is detected from H_2_DCFDA, the CellROX Deep Red evidently localizes into mitochondria (Figure 10). Evaluation of individual subpopulations showed significant increase of CellROX signal from CellROX‐high/H_2_DCFDA‐low population after Venetoclax treatment (Figure 10). Simultaneous labeling with probes for mitochondrial membrane potential showed moderate hyperpolarization of this population, whereas the CellROX‐low/H_2_DCFDA‐high cells had depolarized mitochondria (Figure S6). In addition, positive signal of surface Annexin V, a marker of early apoptosis, was detected from a fraction of cells with high H_2_DCFDA fluorescence (Figure S6).

N‐acetyl‐cysteine (NAC), a compound commonly used to protect cells against oxidative stress, partially reduced the Venetoclax effect (Figure S7). Specifically, decreased CellROX MFI and lower proportion of CellROX‐low/H_2_DCFDA‐high subpopulation were observed in cells pretreated with NAC. The Bcl‐2 fragmentation was also reduced. Importantly, a slight increase of NCL level was simultaneously detected. However, the differences evaluated by immunoblot did not reach statistical significance.

**FIGURE 11:**
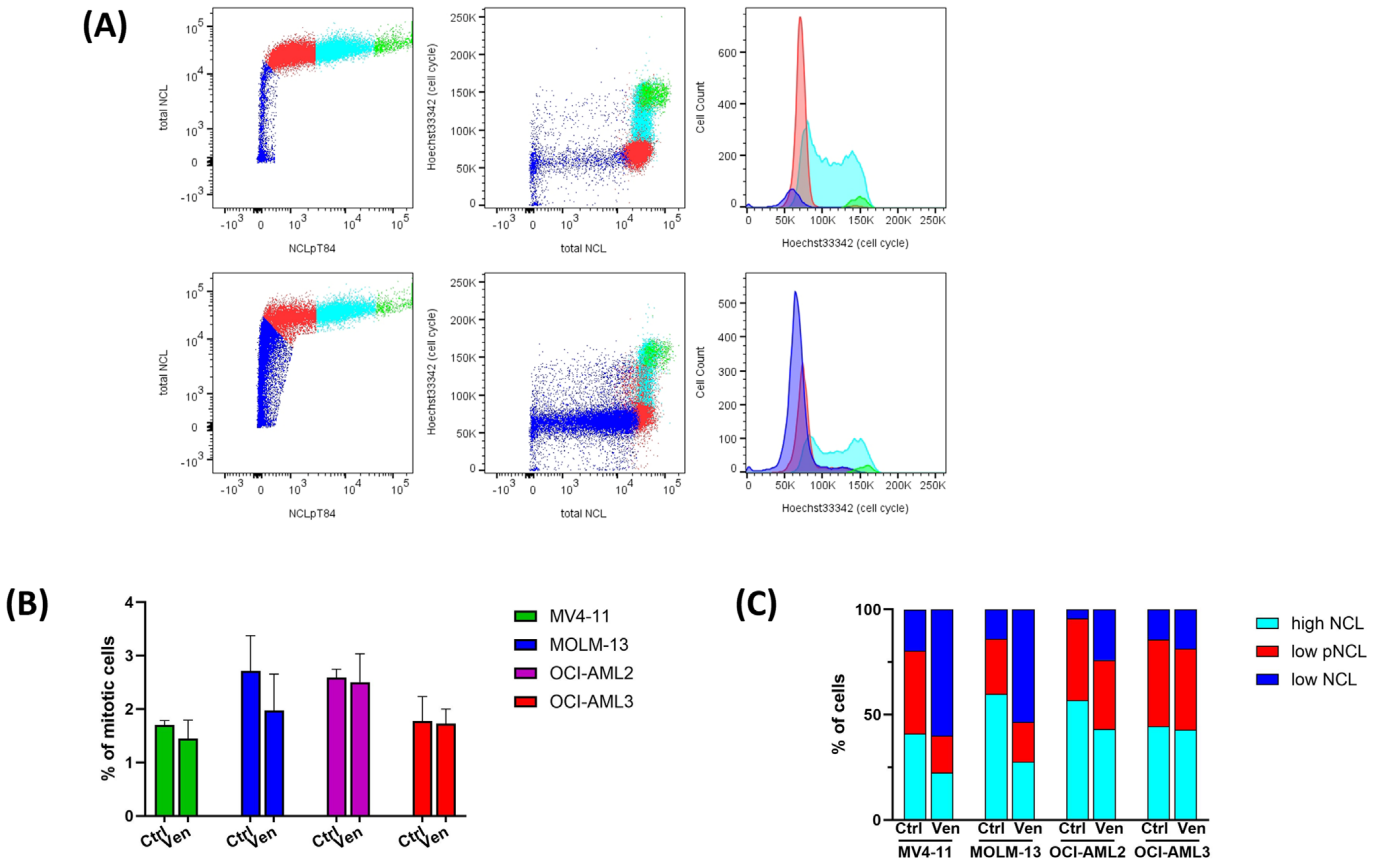
PFA/MetOH‐fixed cells immunostained for total and phosphorylated NCL and for DNA content (Hoechst33342). (A) Representative graphs from untreated (upper row) and 0.5 μM/20 h Venetoclax‐treated (lower row) MOLM‐13 cells. Subpopulation of mitotic cells (very high phospho‐NCL, green), cells with both high NCL level and phosphorylation (cyan), high NCL level/low phosphorylation (red) and both low NCL level and phosphorylation (blue) are marked. Histograms illustrate cell cycle distribution in individual populations. (B) Fraction of mitotic cells in untreated (Ctrl) and Venetoclax‐treated (Ven) AML cell lines. Mean ± SD. (C) Relative representation of fractions determined in (A).

We also examined NCL level in individual cells by immunostaining (Figure 11). Co‐staining for total and phosphorylated NCL together with nuclear DNA staining (Hoechst33342) allowed us to determine changes in the mitotic (high Hoechst33342/very high phospho‐NCL) fraction as well as in signal distribution across the fixed sample (Figure 11). This approach revealed that a fraction of mitotic cells was attenuated in Venetoclax‐treated sensitive cells (Figure 11), which corrected the previous findings from microscopy (Figure 6). Similarly, an enriched fraction of cells with low phospho‐NCL and decreased total NCL signal was detected in Venetoclax‐treated sensitive cells but not in the resistant OCI‐AML3 (Figure 11).

**FIGURE 12:**
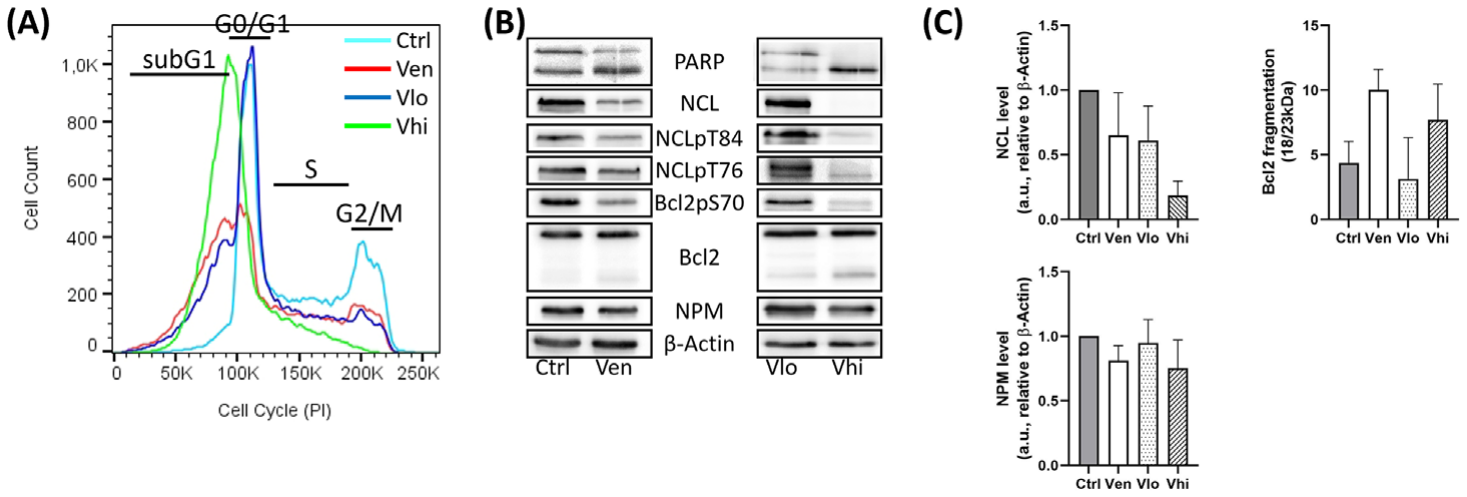
Analysis of subpopulations of 0.5 μM Venetoclax‐treated MOLM‐13 cells sorted according to H_2_DCFDA/PI signal. PI‐negative populations with low (Vlo) and high (Vhi) H_2_DCFDA signal were collected and processed for cell cycle analysis and immunoblotting. (A) Cell cycle distribution in control (cyan line) and Venetoclax‐treated (red line) cells, and in Vlo (blue) and Vhi (green) sorted subpopulations. (B) Apoptosis characteristics and NCL level and phosphorylation in untreated (Ctrl) and Venetoclax‐treated (Ven) cells (left) and in Vlo and Vhi sorted subpopulations (right), representative immunoblots. (C) Statistical evaluation of selected protein differences in samples defined in (B). Mean ± SD values from four experiments are plotted.

Flow‐cytometric sorting of the PI‐negative population according to H_2_DCFDA signal revealed considerable enrichment of subG1 cell cycle phase in the H_2_DCFDA‐high population (Figure 12), which is in agreement with previous fixed cells analysis (Figure 11). Contrary to this, a substantial fraction of cells in G2/M phase was found in the H_2_DCFDA‐low population of Venetoclax‐treated cells, which corresponds with the presence of mitotic cells shown in Figure 6, as well as with results from flow‐cytometric analysis of immunostained cells (Figure 11). Immunoblotting further confirmed decreased NCL level and phosphorylation, and Bcl‐2 dephosphorylation and fragmentation in the sorted H_2_DCFDA‐high population (Figure 12). Importantly, much lesser attenuation of another nucleolar protein nucleophosmin (NPM) was detected, indicating that the NCL changes are not caused by nucleolar degradation.

Altogether, these results confirmed that the H_2_DCFDA‐positive population contains cells directing to apoptosis. Concurrently, a subpopulation of relatively resistant cells occurs, responding to Venetoclax treatment by mitochondria hyperpolarization and induction of ROS detectable with CellROX probes, localizing in mitochondria. Increasing mitochondrial activity induced in these cells likely represents a mechanism of partial Venetoclax resistance even in a relatively homogeneous cell sample (i.e., the cell line).

To deeper investigate the mechanism observed in AML cells, we examined the time‐course of CellROX/H_2_DCFDA/PI signal in the lymphoma cell line HBL‐2, which is highly sensitive to Venetoclax (Figure S8). In this cell line, even 100 nM Venetoclax induced the changes more rapidly, which allowed us to track populations' redistribution. Similarly to AML cells, the originally uniform CellROX‐high/H_2_DCFDA‐low population gradually shifted into the CellROX‐low/H_2_DCFDA‐high subpopulation (Figure S8A). In addition, a double negative population occurred. Concurrently, CellROX Deep Red MFI from the “resistant” population increased during the first hours (Figure S8A), but subsequently this subpopulation almost completely disappeared, indicating high efficacy of Venetoclax treatment, according to this cancer type treatment statistics [43]. Immunoblot analysis confirmed these findings, as caspase‐3 activation, and PARP and Bcl‐2 fragmentation also occurred within hours after Venetoclax addition (Figure S8B). Importantly, although the NCL level and phosphorylation decreased in these cells after short Venetoclax exposition, the level of NPM stayed unchanged, similarly to AML cells (Figure 12). Analysis of Annexin V surface exposition revealed a substantial fraction of Annexin V‐positive cells in both CellROX‐low/H_2_DCFDA‐high and the double negative subpopulations (Figure S8C).

### 3.5 Subtoxic Venetoclax Dose Causes Transient Effect in Sensitive Cells

**FIGURE 13:**
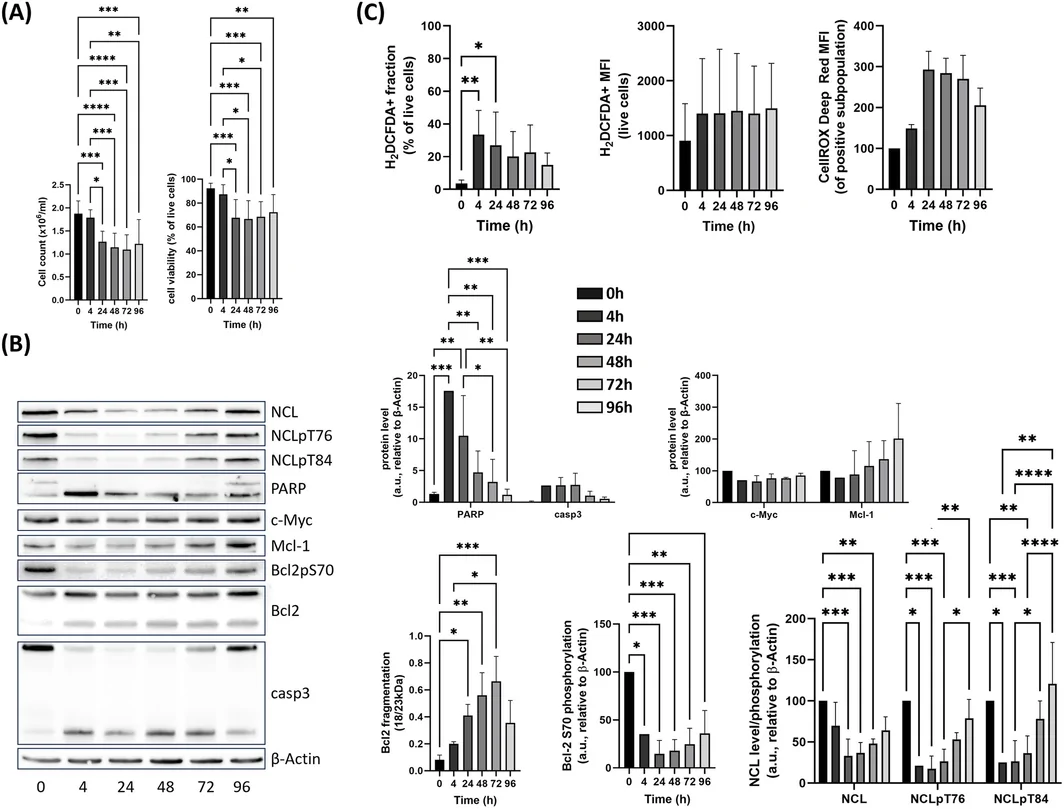
Time evolution of characteristics of MV4‐11 cell line treated with 100 nM Venetoclax in intervals up to 96 h: (A) cell viability and concentration, (B) level of apoptosis‐related proteins and NCL phosphorylations, and (C) frequency of H_2_DCFDA‐positive cells and H_2_DCFDA MFI of live cells, and CellROX Deep Red MFI of CellROX Deep Red‐high/H_2_DCFDA‐low subpopulation. Mean values are plotted with ±SD. Data were processed by ANOVA with multiple comparisons and *p*‐values calculated from at least three repeated experiments. *p < 0.05, **p < 0.01, ***p < 0.001, ****p < 0.0001.

To further study the time course of Venetoclax action and its selective effect, we treated the most sensitive AML cell line, MV4‐11, with 100 nM Venetoclax for up to 96 h. Viability, proliferation, apoptosis, NCL levels, and ROS production were monitored during this time period. Figure 13 shows the transient effect of the subtoxic concentration on all the monitored parameters, with a maximum reached after 24–48 h treatment. We suppose that a sensitive cell subpopulation characterized by high H_2_DCFDA/low CellROX signal has been cleared during this time, followed by a recovery in the remaining cells. These results highlight the importance of carefully balancing Venetoclax dosing to avoid leukemic cell recovery after treatment.

## 4 Discussion

Doxorubicin, a component of intensive AML chemotherapy, caused marked proliferation arrest and loss of viability, whereas Decitabine, a HMA used primarily for patients unfit for intensive care, induced transient growth arrest with minimal impact on cell viability. Venetoclax, recently approved for combination therapy with HMAs [44], exhibited variable efficacy across AML cell lines, reflecting differences in the expression of its primary target Bcl‐2 and its functional alternative Mcl‐1.

Common apoptotic markers, PARP cleavage and caspase‐3 activation, confirmed the proliferation and viability results and enabled classification of patient samples into two groups according to their sensitivity to Venetoclax. Besides PARP and caspase‐3, we analyzed Bcl‐2 fragment related to caspase‐3 activity [45], which provided another independent marker of apoptosis induction. All results corresponded to each other. Bcl‐2 phosphorylation at Serine 70 (Bcl‐2pS70), previously reported to associate with Bcl‐2 activity [46] was used as an indicator of Bcl‐2 inhibition. Attenuation of Bcl‐2pS70 reflected Venetoclax sensitivity, but it was also observed after Doxorubicin treatment, suggesting retrograde signaling triggered by Doxorubicin‐induced DNA damage.

Cell cycle analysis showed considerable S‐phase arrest following Doxorubicin treatment, which correlates with pronounced NCL dephosphorylation at mitosis‐related Threonine sites. In our previous work [47] we described a similar effect induced by the p53 activator RITA, which caused a dramatic decrease in the G2/M fraction and launched apoptosis in the sensitive cell line MV4‐11. That study provided detailed characterization of the activity and distribution of selected phosphorylation forms of nucleolar phosphoproteins NPM and NCL during the cell cycle showing that T76 and T84 are phosphorylated exclusively during premitotic and early mitotic phases [47]. Similarly, a slight increase in the S‐phase fraction after Venetoclax treatment in the current study might partially contribute to the overall NCL dephosphorylation, as the frequency of mitotic cells with a high phospho‐NCL level was also lowered, although there were still numerous mitotic cells detectable by confocal microscopy. Moreover, the total NCL level decreased in Venetoclax‐treated samples, which reduced the number of phosphorylatable molecules and contributed to the attenuated phospho‐NCL level as examined by immunoblot.

NCL phosphorylation during cell cycle is known to be regulated by kinases, in particular by cyclin‐dependent kinases cdk1 and cdk2 [33, 34] and casein kinase CKII [32]. However, our results did not show active participation of these kinases in the NCL regulation, as the phosphorylation of them changed inconsistently. In our samples, the only detectable kinase alteration was cdk‐Y15 dephosphorylation. Nevertheless, the Y15 phosphorylation is referred to inhibit the cdk1 activity [48], contrary to the NCL dephosphorylation detected in our experiments. CKII‐mediated NCL phosphorylation occurs at N‐terminal Serine residues [32] and is therefore unrelated to changes observed at T76 and T84. However, the cdks operate in complexes with different cyclins, and their activity is regulated in multiple ways. During the Doxorubicin treatment, stabilized p53 induces the p21 that further blocks the cdk2 and causes the cell cycle arrest leading to the disappearance of the mitotic cell fraction. In the case of Venetoclax‐induced NCL phosphorylation changes, we suppose that they are caused coincidentally with several factors, including lowered frequency of mitotic cells and decreased level of total NCL.

Mcl‐1 is an antiapoptotic protein frequently compensating Bcl‐2 inhibition [11]. Upregulated Mcl‐1, either inherent or therapy‐related, is often found in cells resistant to BH3 mimetics [49, 50, 51]. Consistent with this, the Venetoclax‐resistant OCI‐AML3 cell line exhibited high basal Mcl‐1 level, which further increased upon Venetoclax treatment. A similar trend was observed in resistant primary cells. Conversely, the reduction of Mcl‐1 level accompanied the Bcl‐2 inhibition in all Venetoclax‐sensitive cells. Interestingly, analogous changes in Mcl‐1 level according to Venetoclax sensitivity were detected in Doxorubicin‐treated cells, indicating common resistance mechanisms based on basal expression levels of Bcl2‐family proteins. Indeed, the Bcl‐2/Mcl‐1 ratio was low in untreated cells of the resistant cell line OCI‐AML3 in comparison with the values from sensitive cells. Similarly, Venetoclax‐resistant primary cells had a lower basal Bcl‐2/Mcl‐1 ratio, although the difference did not reach statistical significance due to one aberrant sample and low patient number. On the other hand, we did not find any correlation between NCL level and sensitivity to Venetoclax. Decitabine effect on Mcl‐1 varies from slight decrease or no change in sensitive cells, to moderate increase in resistant ones. Accordingly, a slight increase of Mcl‐1 induced by Decitabine has been previously described in other myeloid leukemia cell lines [52]. A similar trend as for Mcl‐1 was observed also for the transcription factor c‐Myc, although all these changes were statistically insignificant and should be verified at a larger sample set. Importantly, recent work reported that NCL inhibition with novel aptamer APTA‐16 sensitized AML cells to Venetoclax through downregulation of Mcl‐1 [53].

Venetoclax is considered independent of p53 status and consistently with this we observed no alterations in p53 levels in Venetoclax‐treated cells. However, the Doxorubicin‐induced activation of p53 and its downstream target p21 were markedly attenuated by Venetoclax in sensitive cells. Consequently, p53‐independent p21 induction appeared after 24 h Decitabine treatment in sensitive cells, although this change likely had no effect on cell cycle distribution.

Bcl‐2 inhibition by Venetoclax was not accompanied by corresponding changes in BCL2 transcript level, which is in accordance with the mechanism of Venetoclax action. Importantly, neither NCL deregulation occurs at transcript level, suggesting involvement of some posttranslational or epigenetic mechanisms. Interestingly, Thomalla et al. [14] recently reported increased methylation of BBC3 promotor leading to downregulation of Puma at both mRNA and protein levels in CLL cells resistant to Venetoclax. Co‐treatment with HMA Azacytidine restored Puma protein expression in resistant cells but had an insignificant effect in Venetoclax‐sensitive cells [14]. A similar mechanism may elucidate increased Puma transcript in the sensitive AML cells observed in our experiments, although no corresponding change at the protein level was detected. In fact, the decrease of Puma protein level was even more evident in Venetoclax‐sensitive samples.

NCL is known to regulate BCL2 transcription through interaction with its promoter region [37, 54]. However, Venetoclax‐induced Bcl‐2 inhibition is caused mainly by interference with its BH3‐binding site and does not manifest by significant downregulation of the BCL2 transcript. Moreover, while Bcl‐2 fragmentation and dephosphorylation were observed within hours of treatment, the NCL level attenuation occurred as late as after 24 h of treatment suggesting that NCL deregulation is unlikely to associate with its role in the BCL2 transcription.

ROS production caused by Venetoclax has already been reported previously [19], however, our study provides evidence that this effect is linked to distinct cell subpopulations, characterized by a high cellular/mitochondrial ROS content. The cellular ROS monitored by H_2_DCFDA probe increases in a population that gradually enlarges during several hours after drug addition, and is characterized by markers of oncoming apoptosis, such as mitochondrial membrane depolarization, Bcl‐2 and PARP fragmentation, a high proportion of cells in subG1 phase, and Annexin V positivity. Contrary, the increase of mitochondrial ROS examined by CellROX probe correlates with mitochondria hyperpolarization and absence of signs of apoptosis. The gradual increase of the mean CellROX signal in the less sensitive cell subpopulation is well documented in HBL‐2 cell line, where changes occur rapidly and their monitoring is thus feasible. Using flow cytometry, we cannot determine whether this increase is caused by ROS generation in each individual cell or by a sequential shift of cells with less ROS content to a more sensitive subpopulation. Preliminary microscopy results of single‐cell observation indicate continuous CellROX fluorescence increase in individual cells; however, due to limits of the staining method, this approach will require validation by another method, e.g., with redox sensitive fluorescence protein constructs. At later time points, the CellROX‐low/H_2_DCFDA‐high subpopulation progresses to cell death, as confirmed by PI‐positivity. A sensitive subpopulation was also detected in Doxorubicin‐treated samples. However, it occurred later, and increased H_2_DCFDA fluorescence intensity indicating cellular ROS production was detected even in the less sensitive subpopulation. Doxorubicin‐induced ROS generation is widely described [25, 55, 56] and served mainly as a positive control in this study. Contrary, a variable ROS response has been reported after Decitabine treatment by others [57, 58, 59]. In our earlier work [52] we found that Decitabine‐induced ROS in p53‐null AML cell line HL‐60 occurred in a subpopulation with depolarized mitochondria. The presence of a sensitive subpopulation even in relatively homogeneous samples of cell lines suggests that the sensitivity may be associated with the cell cycle. Further extensive study is required to characterize not only cell cycle variations but also the differences between the populations with high/low ROS on the protein level, with special emphasis on the NCL expression. We also tried to directly correlate CellROX signal with NCL in fixed cells; nonetheless, despite the manufacturer's declarations, none of the CellROX probes (Green, Orange, Deep Red) retained sufficient fluorescence signal after fixation with formaldehyde. Only indirect correlation was thus possible, supposing that subpopulations highly enriched in Venetoclax‐treated samples are identical. With this assumption, the low‐NCL/pNCL population in fixed samples corresponds with the CellROX‐low/H_2_DCFDA‐high population in live cells. This was confirmed by immunoblots of sorted cell subpopulations, where very low levels of NCL protein and its phosphorylation were detected in this sensitive subpopulation. Sorting also proved increased fraction of cells in subG1 phase in the CellROX‐low/H_2_DCFDA‐high population.

We tested several ROS scavengers and NCL inhibitors to manipulate oxidative status or NCL levels in our cells. However, AML cells seem to hardly respond to any of these agents. The ROS scavengers (NAC, sodium pyruvate, D‐mannitol) showed high toxicity for the AML cells in relatively low concentrations. Moreover, Mlejnek et al. [60] showed that in AML cells, NAC induced ROS instead of their scavenging and that it potentiated the cytotoxic effect of several drugs, which highlighted the need for caution when using it in AML cell experiments. Simultaneously, radical scavenging is only one of the multiple effects exerted by NAC, and its antioxidant activity is largely indirect and depends on several factors, including the availability of endogenous cysteine sources [61]. In our experiments, Venetoclax‐sensitive cells pretreated with 1 mM NAC exhibited a partial ROS reduction, a decreased proportion of CellROX‐low/H_2_DCFDA‐high subpopulation, and lower Bcl‐2 fragmentation together with slightly higher NCL level compared with cells treated with Venetoclax alone. These results witness partial cell protection provided by NAC. However, because the NAC‐induced recovery was only partial, the dynamic range for detecting the difference on immunoblots was not sufficient to reach statistical significance. Two available NCL inhibitors (N6L, AS1411) were designed to target minor NCL fraction localized at the cell surface, and they likely were not able to affect overall NCL characteristics. Moreover, we detected Atto425‐labeled AS1411 inside the cells, where it colocalized with lysosomes, suggesting its rapid effective degradation. Genetic manipulations likely might overcome this obstacle, but unfortunately, AML cells are hard to transfect. Using an easily transfectable model system, e.g., HeLa or HEK‐293 T cells, is also impossible due to very low Bcl‐2 expression, and thus the Venetoclax resistance. All relevant approaches will therefore require careful optimization first.

We did not bring any evidence on causality between NCL regulation and ROS generation. Nonetheless, alongside coincidence of small changes induced by NAC, there are some indications in the literature that there can be an association. In particular, excess of ROS production induces nuclear response in the form of retrograde signaling. During oxidative stress, NCL can relocate from the nucleolus to nucleoplasm and cytoplasm [29, 62], interacts with stress‐responsive factors [63] or activates genes involved in antioxidant defense and mitochondrial function [30, 64, 65, 66]. Specifically, Venetoclax was found to disrupt activation of NRF2/KEAP1 pathway, an important component of the antioxidant system, likely by interfering with Nrf2 nuclear translocation and promoting its degradation [22]. Although the exact mechanism is not fully elucidated, considering the NCL's role in chromatin accessibility for Nrf2 target genes, lowered NCL level and activity (phosphorylation) might contribute to decrease of Nrf2 activity and, consequently, to failure of antioxidant defense.

Highly sensitive lymphoma cell line HBL‐2 response was further examined to better understand the Venetoclax‐induced changes. In this cell line, all alterations were observed within several hours. Flow‐cytometry experiments confirmed dynamic changes of cellular and mitochondrial ROS content, identifying additional population characterized by both low CellROX and low H_2_DCFDA signal together with PI negativity. This double negative population occurs together with the H_2_DCFDA‐high one and there is not clear whether it represents a parallel or sequential event during the Venetoclax action, as the distribution of Annexin V signal is comparable in both these subpopulations. Immunoblot confirmed rapid NCL dephosphorylation and degradation while the NPM level remained unchanged. Simultaneously, apoptotic markers such as caspase‐3 activation, and PARP and Bcl‐2 fragmentation are obvious as soon as 2 h after 100 nM Venetoclax addition.

Finally, partial sample recovery after longer (> 24 h) Venetoclax exposure was observed during analysis of concentration and time dependence of Venetoclax‐induced changes in AML patient cells. Long‐term monitoring of subtoxic Venetoclax dose‐treated cells proved partial sample recovery in almost all parameters tested in this study. Specifically, proliferation and viability, apoptotic markers, NCL level and phosphorylation as well as changes in ROS representation peaked at 24–48 h after Venetoclax addition and further remained unchanged or directed back to the initial values. This is in accordance with our findings that Venetoclax treatment identifies two distinct populations with different responses, and that transient increases of mitochondrial ROS together with transient mitochondrial membrane hyperpolarization might protect cells from Venetoclax‐induced apoptosis. These findings are particularly important for BH3‐mimetics therapy adjustment and modifications.

## 5 Conclusion

The Bcl‐2 inhibitor Venetoclax is clinically used to treat patients with CLL and selected cases of AML. However, careful monitoring of blood cell count aberrations is necessary due to the drug's adverse side effects. Here, we describe a previously unknown effect of Venetoclax on the level and activity of the nucleolar phosphoprotein NCL, which complements the general mechanism of Venetoclax action. Importantly, we report the presence of cell subpopulations with varying sensitivity to Venetoclax, reflected in altered responses of the antioxidant defense system. We propose that, after the sensitive population with elevated ROS detected by H_2_DCFDA is eliminated, the remaining cells undergo recovery. This likely contributes to the limited efficacy of Venetoclax monotherapy in AML. Our findings highlight the need for optimized Venetoclax regimens to prevent leukemic cell survival and relapse.

## Author Contributions

Conceptualization: Kateřina Wolfová and Barbora Brodská. Methodology: Barbora Brodská. Validation, Kateřina Wolfová, Petra Otevřelová, and Barbora Brodská. Formal analysis: Barbora Brodská. Investigation: Kateřina Wolfová, Petra Otevřelová, and Barbora Brodská. Resources: Barbora Brodská. Data curation: Barbora Brodská. Writing – original draft preparation: Barbora Brodská. Writing – review and editing: Kateřina Wolfová, Petra Otevřelová, and Barbora Brodská. Visualization: Kateřina Wolfová and Barbora Brodská. Supervision: Barbora Brodská. Project administration: Barbora Brodská. Funding acquisition: Barbora Brodská. All authors reviewed the results and approved the final version of the manuscript.

## Funding

This work was supported by the Ministry of Health of the Czech Republic, Development of the Research Organization (IHBT‐00023736), the project for conceptual development of the research organization.

## Ethics Statement

All patients included in the study provided their written informed consent to the use of their biological material for research purposes. All experimental procedures concerning primary cells were anonymized and followed the ethical standards of the responsible committees on human experimentation and with the Helsinki Declaration of 1975, as revised in 2008. The study was approved by Ethical Committee of IHBT.

## Conflicts of Interest

The authors declare no conflicts of interest.

## Supporting information


**Table S1:** AML patients characteristics.

**Figure S1:** Effect of 1 μM Doxorubicin (Doxo), 0.5 μM Venetoclax (Ven), 1 μM Decitabine (DAC), or their combinations and their combinations on proliferation (left) and viability (right) in individual AML cell lines. Mean values are plotted with ±SD.
**Figure S2:** Effect of 24 h treatment with 1 μM Doxorubicin (Doxo), 0.5 μM Venetoclax (Ven), 1 μM Decitabine (DAC), or their combinations on kinase activity in AML cell lines. Representative images of CKII, CKIIpT360/S362, cdk1 and cdk2 levels, and cdk inhibiting (Tyrosine 15, Y15) and activating (Threonine 160/161) phosphorylations. β‐Actin serves as a loading control.
**Figure S3:** (A) 0.5 μM Venetoclax‐induced changes of BCL2, NCL, and BBC3 mRNA levels. Data are presented as log of change relative to control (untreated) sample. Mean values are plotted with ±SD. (B) Effect of 24 h treatment with 1 μM Doxorubicin (Doxo), 0.5 μM Venetoclax (Ven), 1 μM Decitabine (DAC), or their combinations on AML samples: representative images from immunoblot.
**Figure S4:** Time evolution of ROS production in samples treated for 3, 6, and 24 h with 1 μM Doxorubicin (Doxo), 0.5 μM Venetoclax (Ven), 1 μM Decitabine (DAC) or their combinations. Fraction of ROS‐positive cells (left) and mean of H_2_DCFDA fluorescence intensity relative to control (MFI, right) were evaluated. Asterisks mark statistical significance of difference against appropriate control sample. Mean values are plotted with ±SD. Data were processed by Two‐way ANOVA with multiple comparisons and *p*‐values calculated from at least three repeated experiments. *p < 0.05, **p < 0.01, ***p < 0.001, ****p < 0.0001.
**Figure S5:** Comparison of CellROX probes signal from untreated (cyan) and 0.5 μM Venetoclax‐treated (red) cells. (A) Representative graphs document two distinct populations induced by Venetoclax in sensitive MOLM‐13 cells but not in resistant OCI‐AML3 cell line. (B) MFI of CellROX variants from Venetoclax‐treated cells relative to control in whole sample (left) and in CellROX‐positive subpopulation (right). Red line marks ratio = 1 indicating no change between control and Venetoclax‐treated sample. Mean ± SD represent values from technical triplicates.
**Figure S6:** (A and B) Co‐staining of control (cyan) and 0.5 μM Venetoclax‐treated (red) MOLM‐13 cells for ROS (CellROX Deep Red) and mitochondrial membrane potential (A: TMRM, B: JC‐1). (C and D) CellROX Deep Red, H_2_DCFDA and PE Annexin V distribution in 0.5 μM Venetoclax‐treated MOLM‐13 cells. CellROX Deep Red‐high/H_2_DCFDA‐low (red) and CellROX Deep Red‐low/H_2_DCFDA‐high (green) subpopulations have different signal from Annexin V (C). Annexin V‐positive (orange) cells are mostly CellROX Deep Red‐low/H_2_DCFDA‐high while Annexin V‐negative population (blue) contains both CellROX Deep Red‐high/H_2_DCFDA‐low and CellROX Deep Red‐low/H_2_DCFDA‐high cells (D).
**Figure S7:** Effect of 1 mM N‐acetyl‐cysteine (NAC) pretreatment on 0.5 μM Venetoclax effect in MOLM‐13 cell line. (A) CellROX Deep Red MFI of CellROX Deep Red‐high/H_2_DCFDA‐low subpopulation. (B) proportion of CellROX Deep Red‐high/H_2_DCDA‐low subpopulation in PI‐negative cells. (C) Bcl‐2 fragmentation. (D) NCL level. Mean ± SD, the difference between Venetoclax and NAC + Venetoclax‐treated samples was evaluated using paired *t*‐test, *p < 0.05.
**Figure S8:** Time‐course of 100 nM Venetoclax effect in lymphoma cell line HBL‐2. (A) ROS generation monitored with CellROX Deep Red/H_2_DCFDA co‐staining demonstrates shift from CellROX Deep Red‐high/H_2_DCFDA‐low population (red) to the CellROX Deep Red‐low/H_2_DCFDA‐high (blue) during several hours. Simultaneously, increased CellROX signal from CellROX Deep Red‐high/H_2_DCFDA‐low subpopulation is documented in histograms. (B) Immunoblot image of selected protein levels in cells treated with 100 nM Venetoclax. (C) CellROX Deep Red, H_2_DCFDA and PE Annexin V distribution in Venetoclax‐treated HBL‐2 cells. CellROX Deep Red‐high/H_2_DCFDA‐low (red) and CellROX Deep Red‐low/H_2_DCFDA‐high (green) subpopulations have different signal from Annexin V.

## Data Availability

The authors confirm that the data supporting the findings of this study are available within the article and/or its Supporting Information. Raw data from the immunoblots are openly available in Figshare repository under 10.6084/m9.figshare.33122357.
