# Supplementary material for: Nucleolin Alterations and ROS Production Associate With Sensitivity of AML Cells to Venetoclax
Source: FASEB J. 2026 Sep 26;40(19):e72341. doi: 10.1096/fj.202504934RR (PMC13615419; doi:10.1096/fj.202504934RR)
Supplement: Supplementary file 1 [file FSB2-40-e72341-s002.docx]

Table S1: AML patients characteristics

| Characteristics | (N=33)  No (%) |
| --- | --- |
| Age (median=48) | 20-72 |
| Sex | Male 19/33 (58%)  Female 14/33 (42%) |
| NPM1 mutation | Yes 17/33 (52%)  No 16/33 (48%) |
| FLT3-ITD mutation | Yes 17/33 (52%)  No 16/33 (48%) |
